# Body Fluid-Independent Effects of Dietary Salt Consumption in Chronic Kidney Disease

**DOI:** 10.3390/nu11112779

**Published:** 2019-11-15

**Authors:** Jetta J. Oppelaar, Liffert Vogt

**Affiliations:** Section of Nephrology, Department of Internal Medicine, Amsterdam Cardiovascular Sciences, Amsterdam UMC, University of Amsterdam, Meibergdreef 9, 1105 AZ Amsterdam, The Netherlands

**Keywords:** chronic kidney disease, salt, fibrosis, microcirculation, inflammation, tissue sodium storage

## Abstract

The average dietary salt (i.e., sodium chloride) intake in Western society is about 10 g per day. This greatly exceeds the lifestyle recommendations by the WHO to limit dietary salt intake to 5 g. There is robust evidence that excess salt intake is associated with deleterious effects including hypertension, kidney damage and adverse cardiovascular health. In patients with chronic kidney disease, moderate reduction of dietary salt intake has important renoprotective effects and positively influences the efficacy of common pharmacological treatment regimens. During the past several years, it has become clear that besides influencing body fluid volume high salt also induces tissue remodelling and activates immune cell homeostasis. The exact pathophysiological pathway in which these salt-induced fluid-independent effects contribute to CKD is not fully elucidated, nonetheless it is clear that inflammation and the development of fibrosis play a major role in the pathogenic mechanisms of renal diseases. This review focuses on body fluid-independent effects of salt contributing to CKD pathogenesis and cardiovascular health. Additionally, the question whether better understanding of these pathophysiological pathways, related to high salt consumption, might identify new potential treatment options will be discussed.

## 1. Introduction

Chronic kidney disease (CKD) is a worldwide global health burden. Current international guidelines for CKD treatment recommend dietary and lifestyle modifications to delay progression and to reduce disease-specific mortality in addition to the standard CKD treatment [1]. Regarding salt consumption, these guidelines propose a dietary intake of sodium chloride (NaCl), commonly known as table salt, of less than 5 g daily, which is equivalent to 2 g sodium (Na^+^). The rationale behind this recommendation is based on observations that in CKD patients salt restriction decreases blood pressure (BP) and proteinuria [2,3], i.e., the two principal factors for CKD progression. Salt restriction furthermore prevents glomerular hyperfiltration, and strengthens the renoprotective response to RAAS blockade [3,4,5]. The association between salt consumption and the efficacy of RAAS inhibiting treatment can be explained by the observation that the antiproteinuric effect of RAAS inhibitors is blunted in high salt conditions [6,7]. Additionally, in non-diabetic CKD patients, receiving stable ACE-inhibiting therapy, high salt intake is associated with a higher incidence of end stage renal disease and this effect has shown to be mediated by the waned antiproteinuric effect, independently of BP control [8]. Nonetheless, the exact pathophysiological mechanisms of the beneficial effects of salt reduction are still not fully elucidated. Classically, impaired renal sodium excretion in CKD patients is considered to cause an osmotically driven expansion of the extracellular fluid volume which in turn causes an increase in plasma volume, venous return, cardiac output, and thereby leads to an increase in systemic BP [9,10]. However, in the past several years it has become clear that the effects of salt on renal function are not fully explained by these hemodynamical effects. Studies revealed an association between high salt intake and both immune cell activation and tissue remodeling [11,12]. These mechanisms seem to become increasingly important in our current understanding of the relationship between salt and kidney function in patients with CKD.

## 2. Salt Intake in Relation to Renal Function in CKD Patients

In 1949, it was already described that low salt intake had beneficial effects in CKD patients [13]. However, the strength of the recommendation to lower salt consumption in the current ‘Kidney Disease Improving Global Outcomes’ (KDIGO) guideline is indicated as level 1C, meaning that in time of publication of this guideline clear clinical relevant evidence on the benefits of reducing salt in patients with CKD was lacking. A Cochrane systematic review published in 2005 found that salt reduction in patients with CKD considerably reduced BP and proteinuria [2]. However, this conclusion was based on observational and non-randomized studies with short duration and high quality RCTs were lacking. Recently, Garofalo et al. performed a meta-analysis of randomized clinical trials investigating the effects of dietary salt restriction in CKD [14]. They demonstrated that in patients with CKD stage 1–4 dietary salt restriction per se resulted in improvement of both clinical as ambulatory systolic and diastolic BP and proteinuria. Numerous large clinical trials showed that proteinuria is associated with CKD prognosis, and that reduction of proteinuria lowers the risk of renal events [15]. However, despite its BP and proteinuria-lowering efficacy, thus far evidence of the long-term kidney effects of salt restriction on endpoints such as mortality and long term CKD progression were still lacking. In 2016, the CRIC study was used to analyze the prospective association of urinary sodium excretion with CKD progression and all-cause mortality [16]. This still ongoing multicenter prospective cohort study contained data from 3757 men and women with established CKD ranging from 21 to 74 years of age with eGFR levels between 20 and 70 mL/min/1.73 m^2^. The participants were requested to collect 24-h urine at baseline and follow-up years 1 and 2. The cumulative mean of these three 24-h sodium excretions was used as surrogate for salt intake. After 15,807 person-years of follow-up, a robust and significant relationship between high urinary sodium and CKD progression was showed. This relationship remained significant after adjusting for systolic BP, indicating that high sodium has direct adverse effect on kidney function beyond the increased risk of hypertension. The same cohort also showed a significant association between sodium excretion and cardiovascular disease in the CKD population [17]. The association of high salt intake and worse long-term cardiovascular outcome was also investigated in the PURE cohort [18]. This large-scale epidemiological cohort study of 95,767 individuals aged 35–75 years of the general population of 21 countries reported that the association between sodium intake and the occurrence of major cardiovascular events is not linear, since a significant inverse association was found in the lowest tertile of sodium intake (<4.43 g/day), whilst in the middle tertile (4.43–5.08 g/day) no association was found, and a non-significant positive association was shown in the highest tertile (>5.08 g/day). The PURE study also showed an increased rate of stroke occurrence only among communities with the highest tertile of sodium intake, which were almost all located in China. These findings seem contradictory to the above described protective effects of dietary salt restriction. However, interpretation of the relationship between salt intake calculated from just one morning urine spot sample, as was done in the PURE study, and long term risk factors remains complicated, since not only one but presumably multiyear 24-h urine samples are needed for a correct estimation of sodium intake over the years [19]. Besides increased urinary sodium excretion, acute and chronic changes in salt intake also cause changes in plasma sodium, which may be relevant for the risk of CKD [20]. In a Japanese retrospective 5-year cohort study in 12,041 subjects without diabetes mellitus and/or CKD, it was documented that elevated serum sodium (≥143 mmol/L), corrected for blood urea nitrogen as surrogate for dehydration, may be an independent risk factor for the development of CKD [21]. Direct effects of salt and new concepts in salt homeostasis might provide more insight in the causal pathway of these body fluid-independent effects of high salt intake.

## 3. New Insights in Sodium Homeostasis Set Light on Body Fluid-Independent Effects of High Salt

According to the concept of constancy of the internal environment, as described by Claude Bernard in the 19th century, the function of sodium as the most important regulator of osmolality in the external fluid has dominated our clinical and pathophysiological view of sodium handling. Pursuant to this concept, an abruptly increase of salt consumption leads to sodium accumulation in the extracellular volume, which will be followed by water retention to maintain osmolarity [9,10]. However, due to recent evidence from human and animal studies, this concept, in which sodium is exclusively restricted to the extracellular fluid, is heavily debated. In carefully conducted long-term sodium balance studies in healthy humans, it has been shown that constant high salt intake was not paralleled by the expected weight gain, thus meaning absence of water retention in the body [22]. Furthermore, instead of showing that 24-h sodium excretion matched the dietary salt intake, these carefully conducted sodium balance studies showed that in fixed dietary salt conditions there is day-to-day variability in 24-h sodium excretion, which is accompanied with aldosterone, cortisol, and cortisone fluctuations [23]. As a consequence, long-term total body sodium concentration varied and was independent of total body fluid volume, BP changes, or salt intake, suggesting that sodium was rhythmically buffered and released from the body without simultaneous changes in water content. In keeping with this, an acute sodium balance study performed by our group showed that after a hypertonic sodium infusion, half of the osmotically active sodium ions that were cleared from blood plasma could not be measured in the urine; suggesting storage of sodium ions in an additional compartment, besides the extracellular fluid compartment [24]. Furthermore, in our following study, the opposite was also observed; after a hypotonic fluid load of 20 mL water/kg in 20 min (~1.5 L), it was shown that healthy individuals are able to release sodium from its stores as well [25]. All together, these results indicate the presence of a significant buffer where sodium can both be stored and released. Clearly, these observations have large implications for the interpretation of epidemiological studies reporting on the association between salt consumption and long-term outcomes, that are usually based on estimates that use urine portions or single 24-h samples, and underscored that for the estimation of salt intake multiple 24-h urine samples are needed [19], both in clinical practice, as in epidemiological follow-up studies. Furthermore, these new observations about sodium homeostasis suggest that the association between high salt intake and high BP is possibly more complicated than previously assumed. Regarding the role of tissue sodium storage in this association, several experimental studies show that when tissue sodium storage mechanisms are disrupted, exposure to high salt intake leads to increased blood pressure [26,27]. Nonetheless, this possible beneficial aspect of sodium storage mechanisms in preventing hypertension, the observation that increased skin sodium accumulation occurs in subjects prone for salt-sensitive hypertension implies that it can also be harmful [28,29,30]. The sodium stored in tissue may also directly influence embedded microvessels, since it has been reported that inactive sodium storage could be linked to increased hormonal vasoreactivity, which could increase peripheral resistance and contribute to higher BP [31]. Interestingly, in both salt-sensitive humans and animals, there is evidence that salt-sensitive subjects fail to decrease systemic vascular resistance to a normal extent in response to increases in salt intake [32,33].

In rats, for the first time, the site for sodium accumulation could be identified. It was found that after high dietary salt intake, excessive high sodium concentrations were present in the skin [34]. Glycosaminoglcyans (GAGs), negatively charged sulfated polysaccharides which are abundantly expressed in various tissues, have been identified to be capable of sodium buffering in an osmotically inactive manner [35]. In both animal and human studies, it has been reported that in the skin, known of its large GAG content, high tissue sodium content is accompanied by increased GAGs synthesis, polymerization, and sulfation, which indicates the dynamic capacity of skin sodium storage [35,36]. However, besides the skin, also other tissues, such as blood vessels, the brain, and muscle, have been identified as tissue sodium storage compartments [37,38]. In addition to regulating the capacity of sodium buffering, GAGs may mediate the effects of salt on phagocytes in humans and mice [11,26,39]. Interstitial sodium buffering favors the classical activation of LPS-activated macrophages and enhances their phagocytic activity [11], while the alternative activation of IL4-activated macrophages is limited [40]. Whether activation of circulating macrophages occurs by direct action of sodium, via sodium-induced GAG alterations, or both is not elucidated yet. However, it is becoming increasingly clear that heparan sulfates (type of GAG), both in the microenvironment as well as on the cell membrane of leukocytes, has multiple functions in immune regulation [41]. Furthermore, soluble heparan sulfate fragments are able to activate macrophages in mice [42]. We currently undertake well-controlled sodium intervention studies that will define the crosstalk between GAGs and macrophages in the context of high salt intake (Dutch Kidney Foundation, Kolff grant number 18OKG12).

## 4. The Role of Body Fluid-Independent Effects of High Salt Intake on the Kidney

**Figure 1:**
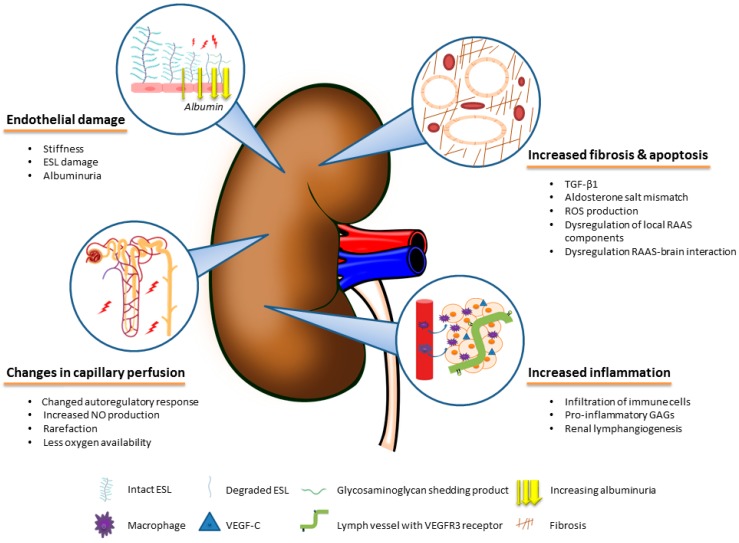
The direct harmful effects of salt on kidney tissue remodeling, kidney microvasculature and renal inflammation.

In both animal and human studies, it has been demonstrated that dietary salt loading directly affect many organs. The deleterious direct effects of dietary salt on the cardiovascular system, bone density, stomach cancer development, and asthma are extensively reviewed elsewhere [43]. Here we focus on the harmful effects of dietary salt to kidney tissue remodeling, kidney microvasculature and renal inflammation, as summarized in Figure 1.

### 4.1. Direct Effects of Salt on Fibrotic Pathways in the Kidney

In both normotensive and hypertensive rats, it was shown that high dietary salt led to widespread glomerular and tubular fibrosis [44]. These fibrotic changes were associated with TGF-β1 overexpression. TGF-β1 is a ubiquitously expressed dimeric cytokine displaying a myriad of biological functions. However, this cytokine has also been shown to play an important role in inducing fibrosis at various sites, including the kidney and blood vessels [45,46]. Additionally, in another animal experiment it was shown that dietary salt increased steady-state levels of mRNA of TGF-β1 in the kidney [47]. Furthermore, the serum TGF-β1 of these animals did not change in response to salt, whilst the urinary excretion did. Thus, indicating the kidney as source of the augmented TGF-β1 excretion. However, inhibiting TGF-β1 as a therapeutic strategy has not yet translated into successful therapy for humans. This may possibly be due to the pleiotropic role of TGF-β1 in generating both fibrotic and kidney protecting effects [48]. All functions of TGF-β1 in the kidney are extensively reviewed elsewhere and behind the scope of this review [48]. Since the expression of TGF-β1 is partly stimulated by aldosterone, it is also important to discuss the pathological interplay between salt and aldosterone in the pathogenesis of kidney fibrosis. Besides the stimulation of TGF-β1, aldosterone in the kidney also activates other profibrotic pathways that are independent of TGF-β1, as summarized in two extensive reviews [49,50]. Briefly, findings of animal studies show that prolonged increased aldosterone levels cause severe glomerular and tubulointerstitial injury [51], particularly in the presence of a high salt diet [52]. In the normal physiological situation, the production of aldosterone by the adrenal gland is suppressed during high sodium conditions, to facilitate renal excretion of sodium excess. Nonetheless, a paradoxical increased mineralocorticoid receptor activation was shown in high salt conditions [53], possibly due to an increased production of tissue aldosterone, which is not always parallel to the circulating level [54]. The importance of salt in the deleterious effects of aldosterone is highlighted by an animal study of Endeman et al., showing that in low salt conditions the association between aldosterone and kidney fibrosis is absent [55]. Additionally, in patients with Gitelman or Bartter syndrome, hyperaldosteronism and low salt conditions are not accompanied with kidney injury [56]. This illustrates that the damaging aldosterone—sodium interplay is only present if serum aldosterone levels are inappropriately high for the sodium status. Since RAAS blockade is known to blunt salt-induced renal injury independent of its blood pressure lowering effect, it is possible that other RAAS associated mechanisms are also involved in the adverse renal effects of high salt intake [57,58,59]. In this context, both dysregulated expression of local of RAAS components as well as a disturbed interaction between RAAS activity in the kidney and the brain may be of importance [60]. The exact pathophysiological pathways behind this association are still subject for research. Furthermore, it has been reported that high salt intake via several pathways stimulates renal damage due to increased production of reactive oxygen species (ROS) in salt-sensitive rats [61] as well as in normal rats [62]. In various tissue types it has been widely established that a relatively high level of ROS leads to redox imbalance, which is associated with cell apoptosis or necrosis [63]. Interestingly, one study in rats on high salt diet reported that hydrogen sulfide (H_2_S) upregulated the expression and antioxidant capacity of one ROS degrading enzyme and thereby improved renal function and renal structural injury. In summary, the direct influence of salt on renal fibrosis covers multiple pro-fibrotic pathways of which some even might amplify each other [64].

### 4.2. Effects of Salt on the Renal Vascular Microcirculation and Endothelium

The microcirculation of the kidney plays a major role in renal oxygen supply and the establishment of plasma filtration, electrolyte exchange, and water reabsorption. Beyond the fact that fluid volume-dependent effects of high salt intake lead to adverse microvascular remodeling, several studies in non-renal tissue show that a high salt diet itself is associated with a reduction of blood vessel density (rarefaction), whilst dietary salt reduction increases vessel number per volume of tissue [65,66,67]. In human kidney tissue, the relevance of a decreased microcirculatory vessel density is illustrated in a study showing a negative correlation between intertubular microcirculatory rarefaction and kidney function [68]. In animal studies, it was reported that rarefaction of the peritubular capillaries is directly correlated with the development of glomerular and tubulointerstitial scarring [69]. However, before disappearance of capillaries, other mechanisms may contribute to high salt-dependent decreased renal tissue oxygenation. In animal studies, it was also shown that high salt affects the autoregulatory response which regulates the vascular tone of the afferent and efferent arterioles of the glomeruli [70,71]. Another important regulator of the tone of kidney blood vessels is nitric oxide (NO), which induces vasodilation of the vascular smooth muscle cells [72]. After high salt intake, increased renal NO synthesis and altered renal nitric oxide synthase (NOS) is reported in several animal studies. However, the mechanisms mediating this response are not yet completely understood [73]. The importance of a decreased renal tissue oxygenation in the pathophysiological pathway of CKD is established in experimental studies [74,75]. In humans, blood oxygenation level-dependent magnetic resonance imaging (BOLD-MRI) has made it possible to measure tissue oxygenation with the use of the paramagnetic properties of deoxyhemoglobin. With this technique, studies have demonstrated that CKD patients have lower renal tissue oxygenation [76,77,78]. However, other studies failed to report differences in renal tissue oxygenation between patients with and without CKD [79,80]. Nonetheless, another study in CKD patients did report, after three years of follow-up, that the lower the oxygenation of the renal cortex, the faster a yearly decline of eGFR is expected [81]. Pruijm et al. showed with BOLD-MRI that in both hypertensive and normotensive individuals that after seven days of a low salt diet, there is an increased oxygenation of the renal medulla and no change in renal cortical oxygenation [82]. This might be of clinical importance since this observation provides an additional argument for the recommendation of salt restriction, although further research in the role of salt restriction in the protection of chronic renal diseases via improved renal oxygenation is needed. Besides influencing oxygenation, it has also been shown in non-renal studies that salt induces stiffness of the endothelial cells and decreased volume of endothelial surface layer (ESL), which is a dynamic GAG-rich layer on the luminal side of the endothelium [83,84]. Regarding kidney function, these are important consequences, since albuminuria is associated with both endothelial stiffness [85] and ESL degradation [86], and diminishes by dietary salt restriction [2,3].

### 4.3. Inflammatory Effects of Salt

Similar to other chronic diseases, CKD is accompanied by low-grade inflammation, which plays a part in CKD progression and outcome [87], however the cause-and-effect relationship between immunity and CKD is still a subject of debate. Renal inflammation occurs with macrophage accumulation and infiltration of inflammatory cells. Among various factors, a high salt diet can cause as well as strengthen the inflammatory milieu in CKD patients [88,89]. When Dahl salt-sensitive rats, which progressively develop hypertension following a high dietary salt intake, are fed a high salt diet for three weeks, increased renal infiltration of macrophages, T-lymphocytes, and B-lymphocytes are measured [88,90]. Furthermore, it was shown in salt-sensitive rats that treatment with immunosuppressive drugs attenuated the renal histological damage during high salt diet [91], providing more evidence for a deleterious role of the immune system in kidney damage during high salt intake. Besides, the attraction of immune cells, also the renal micro-environment, becomes pro-inflammatory after high salt diet. In another animal experiment with normotensive salt-insensitive rats, Hijmans et al. reported that after a high salt diet heparan sulfate, which is the most abundant GAG in the kidney, turned into a pro-inflammatory high sulfated phenotype, mediating inflammation and tissue remodeling [92]. As stated before, in rats it was shown that GAG sulfation in the skin is accompanied with sodium buffering [36]. Interestingly, this study showed that, in contrast to the skin, the kidney was not able to store sodium in high salt conditions. However, there was over time an increasing trend of the expression of podoplanin positive lymph vessels in the kidneys of rats on a high salt diet. Notwithstanding, preliminary results from another more recent study indicate that high salt diet promoted renal macrophage influx in Dahl sensitive rats, which stimulated renal lymphangiogenesis by upregulation of VEGF-C and its receptor VEGFR3 [93]. The clinical consequence of renal lymphangiogenesis was shown in a cohort of 289 CKD patients [94]. In this study, it was reported that higher intrarenal lymph vessel density is associated with more proteinuria, renal fibrosis, interstitial inflammation, and decreased eGFR. A correlation between serum levels of VEGF-C and dietary salt intake has also been reported in CKD patients, supporting lymphangiogenesis in these patients in high sodium conditions [95]. The importance of the production of this growth factor during high salt intake is demonstrated in mice, where administration of exogenous VEGF-C during high salt intake blunted renal fibrosis and decreased the production of oxidative stress markers [96]. Taken all together, these studies underline the complicated mechanisms in which salt through different immunological pathways directly and indirectly influences kidney function.

## 5. Extrarenal Tissue Sodium Storage in CKD

Besides the harmful effects of salt directly on renal fibrotic pathways, renal microcirculation, and renal inflammation, also tissue sodium storage is present in CKD. Specialized ^23^NaMRI studies showed that dermal sodium content as well as muscle sodium content increases with age more progressively in hemodialysis patients than in age-matched controls [29]. Furthermore, it was reported that hemodialysis in these patients resulted in a significantly lower muscle sodium content and a tendency to lower skin sodium compared with controls. In a study using ^23^NaMRI to evaluate tissue sodium concentration in pre-dialysis CKD patients, it was reported that these patients had higher skin sodium concentrations, but no differences were found in sodium muscle concentrations [97]. Moreover, it was found that both salt and water accumulation in these patients was associated with the elevation of serum markers for endothelial activation and of inflammatory pathways. Tissue sodium in type 2 diabetic patients on hemodialysis was even higher when compared to control hemodialysis patients [98]. A German study investigating the skin sodium content in ninety-nine CKD patients (median eGFR of 51 mL/min/1.73 m^2^) measured a wide range of skin sodium levels [99]. The mean skin sodium level did not appear to differ from levels in healthy controls measured in other studies. However, some CKD patients showed concentrations which overlapped with the skin sodium levels found in hemodialysis studies. Another study was also not able to find differences in dermal sodium concentration between kidney patients undergoing transplantation and healthy controls [12], but their results were possibly influenced by the dialysis which part of the kidney patients received before the measurement of skin sodium levels [29]. Nonetheless, Hijmans et al. were able to show that both the skin of pre-emptive and dialyzing patients contained more macrophages [12]. Also, their dialysis patients showed an increased lymphangiogenesis in skin biopsies. In type 1 diabetic patients, which share the trait of salt-sensitivity with CKD patients, our group was also able to show that these patients had higher skin lymphatic microvessel density in low salt conditions when compared to healthy controls [100]. This might reflect a higher skin sodium content in these patients, even in low dietary salt conditions. All studies measuring tissue sodium levels in CKD, however, did not use a sodium intervention, therefore the measured wide ranges of tissue sodium concentrations could possibly be explained by the interpersonal differences in sodium intake. In healthy participants, a randomized controlled trial showed an increased skin sodium content after a high sodium diet for seven days, which correlated with BP, stroke volume, and peripheral vascular resistance [101]. Up until now, such a study has not yet been performed in CKD patients. Additionally, the association between skin sodium storage and outcome has not been studied in detail in CKD patients. Yet, one study showed an association between skin sodium and left ventricular hypertrophy in advanced non-dialyzing CKD patients [99]. Beside sodium buffering in the skin, the ESL also plays an important role in non-osmotic sodium buffering [102]. By ^23^Na nuclear magnetic resonance, it was shown that GAGs in the ESL can reversibly bind to sodium under flow [103]. In CKD patients, elevated shedding products of the ESL, reflecting ESL breakdown, are present [104]. In type 1 diabetic patients, ESL damage is associated with increased BP, which suggests a possible association between ESL volume and BP regulation [86]. Since CKD patients represent a sensitive and vulnerable group because of difficulties in the regulation of sodium and water homeostasis, the clinical relevance of further research into extrarenal sodium regulation and its consequences in these patients is high.

## 6. Non-Osmotic Sodium Buffering as Potential Treatment Target

**Figure 2:**
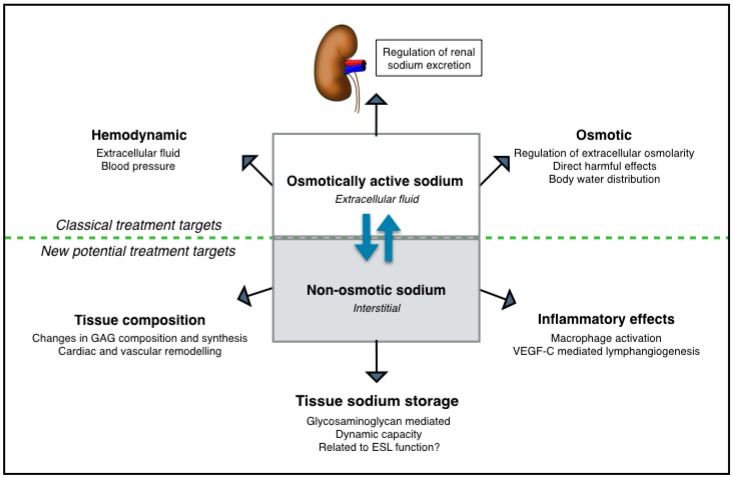
Different mechanisms of sodium handling and their effects on the human body with regard to possible treatment targets (either life-style or pharmacologically mediated). Below the dotted line the mechanisms associated with non-osmotic sodium storage are visualized, for these mechanisms further research into potential treatment targets is necessary.

Beside the clinical importance of gaining more knowledge about sodium homeostasis in CKD, insight in extrarenal sodium handling could also provide possible new treatment targets for this patient group. The effects of extrarenal sodium handling are summarized in Figure 2, and can be divided in both beneficial as well detrimental effects. First, its buffering function during osmotic stress, as induced by a high dietary salt load, might prevent both development of hypervolemia as well as the deleterious effects of high serum sodium levels. In this regard, restoration of the non-osmotic sodium buffering compartment with oral GAG supplementation is of interest. A meta-analysis demonstrated that sulodexide, an oral drug consisting of a highly purified mixture of GAGs, significantly reduced blood pressure in hypertensive subjects, presumably due to neutralization of the negative effects of excessive sodium [105]. A study in stage 3 and 4 CKD patients also showed a small but significant reduction in BP after sulodexide treatment when compared to placebo, however, no additional renoprotective effects of sulodexide were seen [106]. Nonetheless, negative effects of extrarenal sodium buffering may relate to the observation that increased tissue sodium content on ^23^NaMRI are associated with many cardiovascular risk factors and comorbidities [30,107]. It is not fully elucidated how tissue sodium content under these conditions should be interpreted, but strong associations with cardiac and vascular remodeling [99,108], potentially leading to cardiovascular mortality, have been reported. Besides, since increased dermal osmolarity attracts macrophages and favors the pro-inflammatory activation of macrophages, non-osmotic sodium storage may maintain or even increase the chronic inflammatory state in CKD [26,27]. Therefore, extrarenal non-osmotic sodium handling seems to act as a two-edged sword: On one hand, it protects against negative effects of excessive sodium in acute settings, and on the other hand, in conditions of persistent demand, its fortune may reverse by increasing inflammation and the risk of worse cardiovascular outcomes. However, the current treatment strategies including diuretics and dietary salt restriction are mainly focusing on lowering BP levels, increasing urinary sodium excretion, and preventing the direct deleterious effects of salt on several tissues. However, the effect of these strategies on extrarenal sodium handling nor the influence of extrarenal sodium handling on these strategies is not elucidated yet. Therefore, more insight in the pathophysiology of dermal non-osmotic sodium storage in CKD is needed to reveal pathways responsible for the deleterious effects of salt.

## 7. Conclusions

In conclusion, the detrimental effects of salt go beyond extracellular fluid volume-associated effects. Dietary high salt consumption directly induces renal fibrosis, damage of renal microcirculation, and increases inflammation, all independently of changes in BP. Furthermore, recent studies have shed light on new sodium handling pathways which potential roles can be divided in beneficial and deleterious effects, respectively. Non-osmotic sodium buffering seems to act as a two-edged sword: It seems to protect against acute excessive salt load, however, in conditions of persistent high dermal sodium levels, inflammation is increased as well as the risk of worse cardiovascular outcomes. In CKD dermal non-osmotic sodium, buffering seems to differ from healthy controls. However, fundamental knowledge of the consequences of these differences regarding disease progression and the response to dietary salt loading is lacking. Nonetheless, this bears essential consequences, since in clinical practice the treatment strategies are still focusing on extracellular fluid volume-dependent effects of salt. More research is needed to further elucidate the pathophysiological pathways of this mechanism and its consequences in CKD.
